# Cerebellar Exposure to Cell-Free Hemoglobin Following Preterm Intraventricular Hemorrhage: Causal in Cerebellar Damage?

**DOI:** 10.1007/s12975-017-0539-1

**Published:** 2017-06-10

**Authors:** Alex Adusei Agyemang, Kristbjörg Sveinsdóttir, Suvi Vallius, Snjolaug Sveinsdóttir, Matteo Bruschettini, Olga Romantsik, Ann Hellström, Lois E. H. Smith, Lennart Ohlsson, Bo Holmqvist, Magnus Gram, David Ley

**Affiliations:** 1 Pediatrics, Department of Clinical Sciences Lund, Skåne University Hospital, Lund University, BMC C14, SE-221 84 Lund, Sweden; 2 Department of Ophthalmology, Institute of Neuroscience and Physiology, Sahlgrenska Academy, University of Gothenburg, Gothenburg, Sweden; 3 Department of Ophthalmology, Boston Children’s Hospital, Harvard Medical School, Boston, MA USA; 4 MicroMorph Histology Services, Lund, Sweden; 5 ImaGene-iT AB, Medicon Village, Lund, Sweden; 6 Infection Medicine, Department of Clinical Sciences Lund, Lund University, Lund, Sweden

**Keywords:** Intraventricular hemorrhage, Hemoglobin, Haptoglobin, Cerebellum, External granular layer

## Abstract

Decreased cerebellar volume is associated with intraventricular hemorrhage (IVH) in very preterm infants and may be a principal component in neurodevelopmental impairment. Cerebellar deposition of blood products from the subarachnoid space has been suggested as a causal mechanism in cerebellar underdevelopment following IVH. Using the preterm rabbit pup IVH model, we evaluated the effects of IVH induced at E29 (3 days prior to term) on cerebellar development at term-equivalent postnatal day 0 (P0), term-equivalent postnatal day 2 (P2), and term-equivalent postnatal day 5 (P5). Furthermore, the presence of cell-free hemoglobin (Hb) in cerebellar tissue was characterized, and cell-free Hb was evaluated as a causal factor in the development of cerebellar damage following preterm IVH. IVH was associated with a decreased proliferative (Ki67-positive) portion of the external granular layer (EGL), delayed Purkinje cell maturation, and activated microglia in the cerebellar white matter. In pups with IVH, immunolabeling of the cerebellum at P0 demonstrated a widespread presence of cell-free Hb, primarily distributed in the white matter and the molecular layer. Intraventricular injection of the Hb scavenger haptoglobin (Hp) resulted in a corresponding distribution of immunolabeled Hp in the cerebellum and a partial reversal of the damaging effects observed following IVH. The results suggest that cell-free Hb is causally involved in cerebellar damage following IVH and that blocking cell-free Hb may have protective effects.

**Electronic supplementary material:**

The online version of this article (doi:10.1007/s12975-017-0539-1) contains supplementary material, which is available to authorized users.

## Introduction

Cerebral intraventricular hemorrhage (IVH) continues to be a serious complication of preterm birth, resulting in a high incidence of neurodevelopmental impairment, including cerebral palsy and intellectual disability [1]. During the past decades, neurological impairment following very preterm birth has primarily been considered to originate in cerebral white matter lesions [2, 3], but recent findings have also linked neurological deficits of preterm birth to cerebellar abnormalities [4, 5]. Prevalence of cerebellar injury has been described to be as high as 58% in infants with cerebral palsy following IVH and preterm birth [6].

From gestational weeks 20 to 40, the cerebellum undergoes an unparalleled growth with a volumetric increase from approximately 1 to 25 cm^3^ [7]. This rapid growth renders the cerebellum very sensitive to injury [8, 9]. Cerebellar underdevelopment may ensue from a direct cerebellar injury, such as hemorrhage or infarction, or from a secondary effect related to damage at a remote but connected area of the brain [10]. Cerebellar hypoplasia has repeatedly been shown to be associated with supratentorial IVH in very preterm infants and is a potential component in neurological disability [9, 11, 12]. Of note, the severity of IVH is linked to the degree of impaired cerebellar development in preterm infants, with cerebellar volume at term age being inversely correlated with increasing severity of IVH [7].

In clinical studies, MRI at term age shows infratentorial hemosiderin deposits in 70% of preterm infants with IVH and disrupted cerebellar development. The deposits are prominent not only on the cerebellar surface but also on the surface of the brain stem and in the region of the fourth ventricle. This hemosiderin deposition is the most predictive factor for impairment in cerebellar development and thus is suggested as a plausible causal mechanism of cerebellar hypoplasia following preterm IVH [9].

The neurotoxicity of cell-free hemoglobin (Hb) and its metabolites has been reported after intraventricular, intraparenchymal, and subarachnoid hemorrhage (SAH) [13–20]. Cell-free Hb and its metabolites free heme, iron, reactive oxygen species (ROS), and free radicals can be highly damaging to cells, lipids, proteins, and DNA through oxidative modification, fragmentation, and cross-linking [21–23]. Cell-free Hb and its metabolites can induce cytotoxic, oxidative, and inflammatory pathways in the cerebrospinal fluid (CSF) and choroid plexus ependyma leading to tissue damage and cell death following preterm rabbit pup IVH [17–19]. Furthermore, a high accumulation of cell-free Hb in the periventricular white matter has been observed following hemorrhage in the rabbit pup IVH model [20].

In this study, we have completed the first investigation of the exposure of the developing cerebellum to cell-free Hb following preterm IVH and the potentially damaging effect on cerebellar development. Furthermore, we report on the protective effects of the Hb scavenger haptoglobin (Hp) following intraventricular administration. Results show that after IVH, key cell populations of the developing cerebellum are exposed to cell-free Hb, which may be central in the pathophysiological events leading to cerebellar underdevelopment.

## Materials and Methods

### Animals

The study was approved by the Swedish Animal Ethics Committee in Lund. We used the well-established preterm rabbit pup model of glycerol-induced IVH as previously described [24]. The study included 59 rabbit pups from 9 litters delivered at gestational day 29 (full term corresponding to 32 days) [25, 26]. A half-breed between New Zealand White and Lop was used. The pups were delivered by caesarean section after the does were anesthetized with i.v. propofol (5 mg/kg) and with local infiltration of the abdominal wall using lidocaine with adrenaline (10 mg/ml + 5 μl/ml, 20–30 ml). After delivery, the pups were dried, weighed, and placed in an infant incubator set to a temperature of 34–35 °C and ambient humidity. At 2 h of age, the pups were hand-fed with 2 ml (100 ml/kg/day) of kitten milk formula (KMR; PetAg Inc., Hampshire, IL, USA) using a 3.5 French feeding tube and fed every 12 h increasing each meal by 1 ml. At 2 h of age, the pups were injected intraperitoneally with 50% (v/v) sterile glycerol (6.5 g/kg; Teknova, Hollister, CA, USA) to induce IVH. Ultrasound imaging of the brain was performed at 6 h of age to grade the severity of the IVH and detect SAH and daily thereafter using the VisualSonics Vevo 2100 (VisualSonics Inc., ON, Canada) with a MS-550D 40 MHz transducer. Animals with IVH at 6 h were included in the IVH group, and those without detectable IVH at all time points were used as controls (denoted as sham control). The reproducibility and accuracy of high-frequency ultrasound in this animal model have been described previously [24].

### Intraventricular Injections

**Fig. 1:**
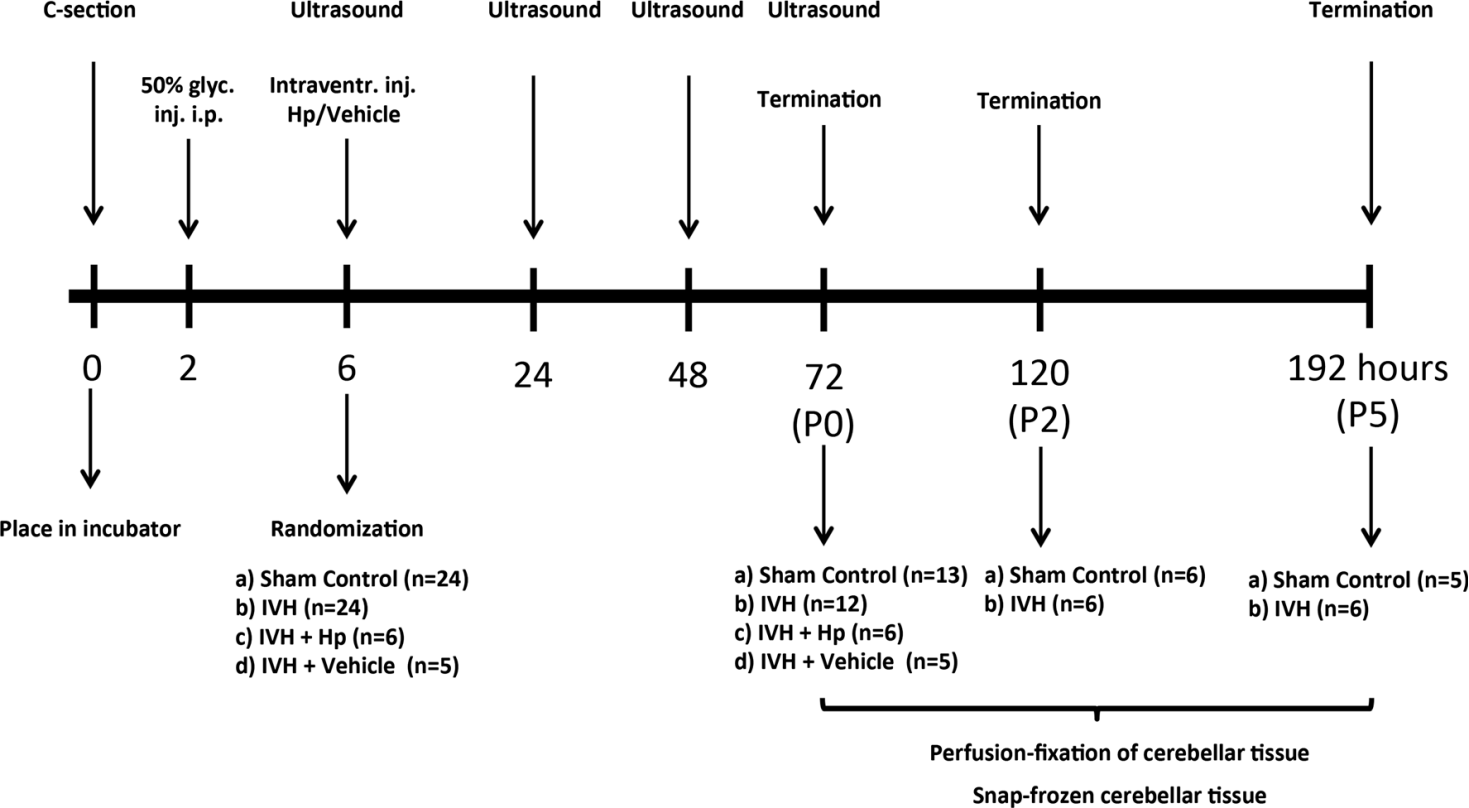
Study outline. A diagram summarizing the experimental procedure. The experiment consisted of the following steps: preterm delivery of rabbit pups by caesarean section, induction of IVH by intraperitoneal glycerol administration, verification of IVH or sham control by the use of high-frequency ultrasound, randomization into study groups, intraventricular administration of Hp or vehicle solution, termination of pups, and collection of cerebellar tissue. For details about each step, see “Materials and Methods”

After the initial ultrasound examination at 6 h of age, pups with IVH (presence of blood within distended lateral ventricles and no sign of parenchymal involvement) were randomized into one of the following three groups: IVH, IVH + Hp, or IVH + Vehicle. Pups in the IVH + Hp and IVH + Vehicle groups received an ultrasound-guided intraventricular injection at 8 h of age of either 20 μl of human Hp (50 mg/ml, Bio Products Laboratory, London, UK) or 20 μl of vehicle solution (9 mg/ml NaCl, Fresenius Kabi, Lake Zurich, IL, USA), using 27 G Hamilton syringes (Hamilton Robotics, Reno, NV, USA). The efficacy and accuracy of this method have been described previously [26]. The animals were euthanized at the following time points: 72 h (P0, corresponding to term-equivalent postnatal day 0), 120 h (P2, corresponding to term-equivalent postnatal day 2), or 192 h (P5, corresponding to term-equivalent postnatal day 5) of age. Cerebellar tissues were sampled and processed as described below. An overview of the study design is given in Fig. 1.

### Tissue Collection and Processing

Following sedation with isoflurane inhalation, perfusion fixation of the brain was performed at P0 and P5 by cardiac cannulation following thoracotomy and infusion of 0.9% saline followed by 4% paraformaldehyde (PFA, buffered with phosphate buffer saline (PBS) 0.1 M, pH 7.4). After completed perfusion, the cerebrum and cerebellum were carefully extracted from the skulls and immersed in 4% PFA for a total of 48 h. SAH was confirmed in all pups with IVH with visible presence of hemorrhagic CSF covering the cerebellar cortex. None of the control pups exhibited macroscopic signs of SAH. A change to fresh PFA was performed after 3–6 h. Thereafter, the tissues were dehydrated, cleared, infiltrated with paraffin, and embedded in paraffin blocks. The cerebellum was sectioned into 4-μm sections (Leica, RM2255 Microtome) in the parasagittal plane at the level of the dentate nucleus and mounted on microscope slides and dried at 37 °C for 12–16 h. None of the cerebellar samples in pups with IVH or in control pups exhibited signs of primary cerebellar hemorrhage. Prior to antibody staining for immunohistochemistry (IHC), the sections were rehydrated, followed by heat-induced antigen retrieval at 90–95 °C for 20 min either in boric acid buffer (pH 8.0) for labeling of Ki67, calbindin, and Iba1 or in citric acid (pH 6.0, with 0.05% Tween 20 or 0.2% Triton X) for 10–20 min for immunofluorescence labeling of Hb and Hp.

### Immunofluorescence Labeling

Immunofluorescence labeling of Hb was performed to investigate the presence and distribution of both encapsulated erythrocytes and cell-free Hb within the cerebellum. Double immunofluorescence labeling of Hb together with human Hp was performed to simultaneously visualize Hb and Hp to elucidate whether the intraventricularly injected human Hp could reach the cerebellar brain regions containing Hb (preferentially the cell-free Hb) in the IVH rabbit pups.

In brief, the immunofluorescence labeling protocol was carried out as described below. Following antigen retrieval, sections were immersed in PBS (2 × 5 min), encircled with silicon (PAP-pen, Sakura, Tokyo, Japan), and then blocked with 1% bovine serum albumin (BSA) in PBS containing 0.05% Triton X (PBST × BSA) for 60 min at room temperature (RT). This step was followed by 16 h of incubation at 4 °C with either one of the primary antibodies or a mixture of the two primary antibodies diluted in PBST × BSA. All antibody incubations were performed in a moisture chamber. Primary antibodies used were against Hb, made in goat (diluted 1:500), and against human Hp, made in chicken (diluted 1:1000), both from GenWay Biotech, Inc. (San Diego, CA, USA) and diluted in PBST × BSA. Sections were then rinsed in PBS (3 × 3 min), followed by incubation for 60 min at RT with one secondary antibody made against goat IgG or with a mixture of secondary antibodies made against goat IgG and chicken IgY (diluted 1:200 in PBST × BSA). The secondary antibodies were both affinity-purified Fab2 fragments for multi-labeling, made in donkey (Jackson ImmunoResearch, West Grove, PA, USA). The anti-chicken IgY was conjugated with Alexa Fluor 488 (AF488) and the goat IgG conjugated with Rhodamine Red (rhodamine). Sections were then rinsed in PBS (3 × 3 min) and incubated in DAPI (0.1 μM, diluted in PBS, Invitrogen, Rockford, IL, USA) for 30 min at RT. After being rinsed in PBS (3 × 3 min), sections were mounted (Fluoroshield, Abcam, England, ab104135) and cover-slipped. All animal groups were always processed together in the same immunolabeling experiment.

### Antibody Control for Immunofluorescence Labeling

**Fig. 2:**
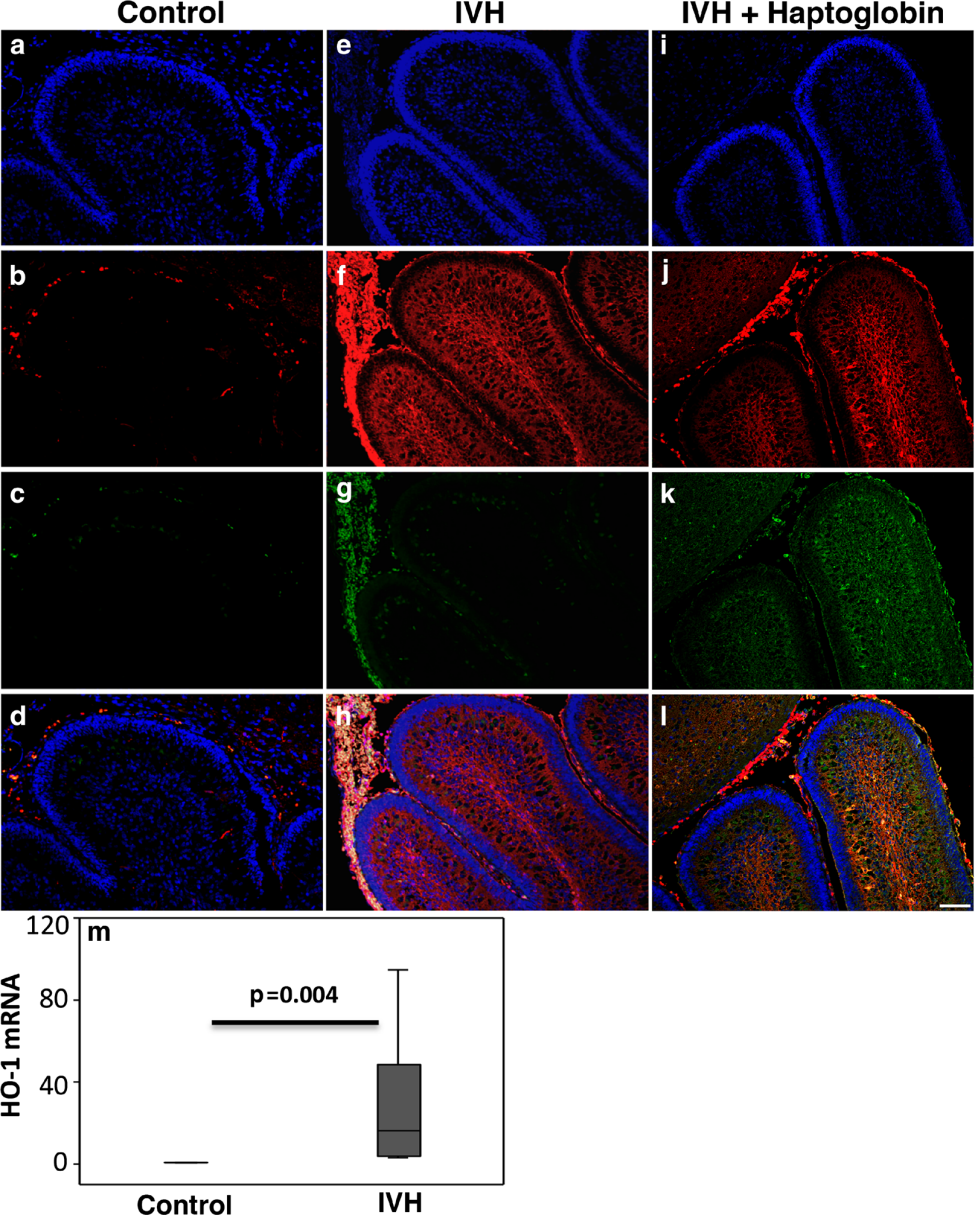
Immunofluorescence labeling of Hb and the administered human Hp. Representative images are from rabbit pups at P0. Images illustrate the detected immunofluorescence labeling, performed by double immunofluorescence labeling of Hb (*red*) and Hp (*green*) together with a DAPI nuclear staining (*blue*), in animals with no IVH (*Control*), in animals with IVH (*IVH*), and in animals with IVH that received human Hp injections (*IVH + Haptoglobin*). Antibody specificity tests showed that the antibodies against Hb and human Hp bound to their true targets (see Fig. 1 in the Data supplement). **a**–**d** Control animal: Images **b** and **c** show the lack of Hb and Hp labeling and the autofluorescence mainly from whole erythrocytes (RBCs) restricted to the subarachnoid space and some blood vessels (**d**). **e**–**h** IVH: In pups with IVH, the Hb labeling (*red*) was extensive, widely distributed in the molecular layer and white matter and to some degree in the EGL. Whole erythrocytes in the subarachnoid space surrounding the cerebellar lobuli were also intensely labeled and gave rise to *green* autofluorescence (**g**), observed as *yellowish* in the merged image (**h**). Hb labeling intermingled with dense nuclear regions (intense DAPI staining) appears as *pink* (*bottom images*). **i**–**l** IVH + Haptoglobin: **j** and **k** show immunofluorescence labeling of Hb (*red*) and human Hp (*green*) following intraventricular injection of Hp at E29. **j** shows the widespread distribution of cell-free Hb (*red*), corresponding to that in IVH animals (**f**), and the domination coexistence of Hp in K (*green*), primarily in the molecular layer, white matter, and the EGL as shown in the merged image (**l**). Hp labeling was scarce in the subarachnoid space (**k** and **l**), in which Hb labeling of RBCs was extensive (**j** and **l**). Thus, the cell-free Hb and Hp are clearly distinguishable from the cell body–associated Hb labeling and autofluorescence. *Scale bar* = 50 μm. **m** HO-1 mRNA expression in the cerebellum was investigated at P0 following IVH*.* Following IVH, heme-degrading protein HO-1 mRNA was upregulated (IVH, *dark gray bar*, *n* = 7) as compared to the controls (*n* = 5). mRNA expression for HO-1 was normalized against GAPDH and is given as fold change. The fold change values were calculated by normalizing against samples from control pups. Results are presented as box plots displaying medians and 25th and 75th percentiles. Differences between no IVH and IVH at P0 were analyzed using the Mann–Whitney *U* test

Antibody specificity tests were performed on parallel sections in all labeling experiments; in these tests, the primary antibodies were excluded from the labeling protocol (Fig. 1 in the Data supplement). This control confirmed that the visualized and documented Hb and Hp immunofluorescence labeling (Fig. 2) was caused by binding of the respective primary antibodies and was not the result of binding of secondary antibodies or autofluorescence. All tested samples showed no Hb or Hp labeling within the cerebellum (see Fig. 1 in the Data supplement). Autofluorescence was solely obtained from whole cell bodies, from erythrocytes/RBCs in the subarachnoid space preferentially, and occasionally from some neuronal cell bodies. Thus, the antibody controls showed that both primary and secondary antibodies bind to their targets in the immunofluorescence labeling protocol applied here, supporting their specific detection of rabbit Hb and human Hp, visualized as extracellular (cell-free) and in whole erythrocytes.

### Analyses of the Distribution of Hb and Its Relation to Hp

The anatomical distribution of the double immunofluorescence-labeled Hb and Hp was analyzed using a wide-field epi-fluorescence microscope (Olympus IX73, Shinjuku, Tokyo, Japan). Analysis of the double labeling was performed by switching between the specific filter sets used for each fluorophore, DAPI for cell nuclei (blue), rhodamine for Hb (red), and AF488 for Hp (green), together with digital image documentation (Olympus DP80). The separate images for each channel (fluorophore) were merged for detailed analyses of double labeling to identify them as co-existing or not (see representative images in Fig. 2). To ensure sole detection of primary antibody binding, i.e., excluding detection of autofluorescence or of nonspecific secondary antibody binding, the detection level (threshold) for each channel was always set from sections with antibody controls (see above) and from sections from control animals that had been taken through the whole labeling protocol. Analyses and digital imaging were performed with the preset detection levels (detection intensities solely from specific labeling) for each channel. The relatively strong autofluorescence from cell bodies, mainly from RBCs, could be clearly separated from the non-cell body-associated, cell-free, and widely distributed Hb in IVH animals and together with Hp in IVH animals that received Hp (see Fig. 2).

### Immunohistochemistry of Cerebellar Development and Reactive Microgliosis

To investigate the effect of IVH on the cerebellum of preterm rabbit pups, IHC labeling against the following antigens was performed: (1) Ki67, to evaluate cellular proliferation; (2) calbindin, to evaluate Purkinje cell development and maturation; and (3) Iba1, to evaluate microglial activation. Qualitative and quantitative analysis at P0, P2, and P5 were performed. Briefly, the protocol was as follows. After antigen retrieval and rinsing in PBS, sections were incubated with primary antibodies (diluted in PBS + 5% normal goat serum, Jackson ImmunoResearch, 005-000-121) for 1 h at RT. Primary antibodies were made against rabbit Ki67 (mouse IgG anti-Ki67, Dako, Copenhagen, Denmark), calbindin (mouse IgG anti-calbindin, DBS, Pleasanton, CA), and Iba1 (rabbit IgG anti-Iba1, Biocare, Concord, CA). Sections were then rinsed in PBS (3 × 2 min). To detect the primary antibody, sections were incubated with either BrightVision rabbit/horseradish peroxidase (HRP) or BrightVision mouse/HRP (DPVR110HRP or DPVM110HRP, both from Immunogen) for 30 min at RT. Sections were then rinsed in Tris (0.05 M, pH 7.6, 3 × 2 min). To visualize the HRP conjugations, sections were incubated with a diaminobenzidine (DAB; 50 mg DAB, Sigma, dissolved in 100 ml Tris buffer, pH 7.6, 3 × 2 min) and 100 μl of hydrogen peroxide (Merck, prepared just prior to incubation) solution was added for 5 min at RT. After rinsing in Tris (3 × 2 min), hematoxylin staining of cell nuclei (Mayers HTX, Bio-Optica) was performed for 5 s, after which the sections were dehydrated and slides were then mounted with coverslips (X-Tra-Kitt, Medite, Burgdorf, Germany). Antibody specificity tests were performed on parallel sections to confirm that the visualized immunostaining was specific for the primary antibodies. In these tests, the primary antibodies were excluded from the labeling protocol (Fig. 2 in the Data supplement). Analysis and image documentation for the results of qualitative and quantitative analysis (see below) of IHC labeling were performed with a bright-field microscope (Leica DMRX), equipped with a digital camera (Leica MC120HD).

**Fig. 3:**
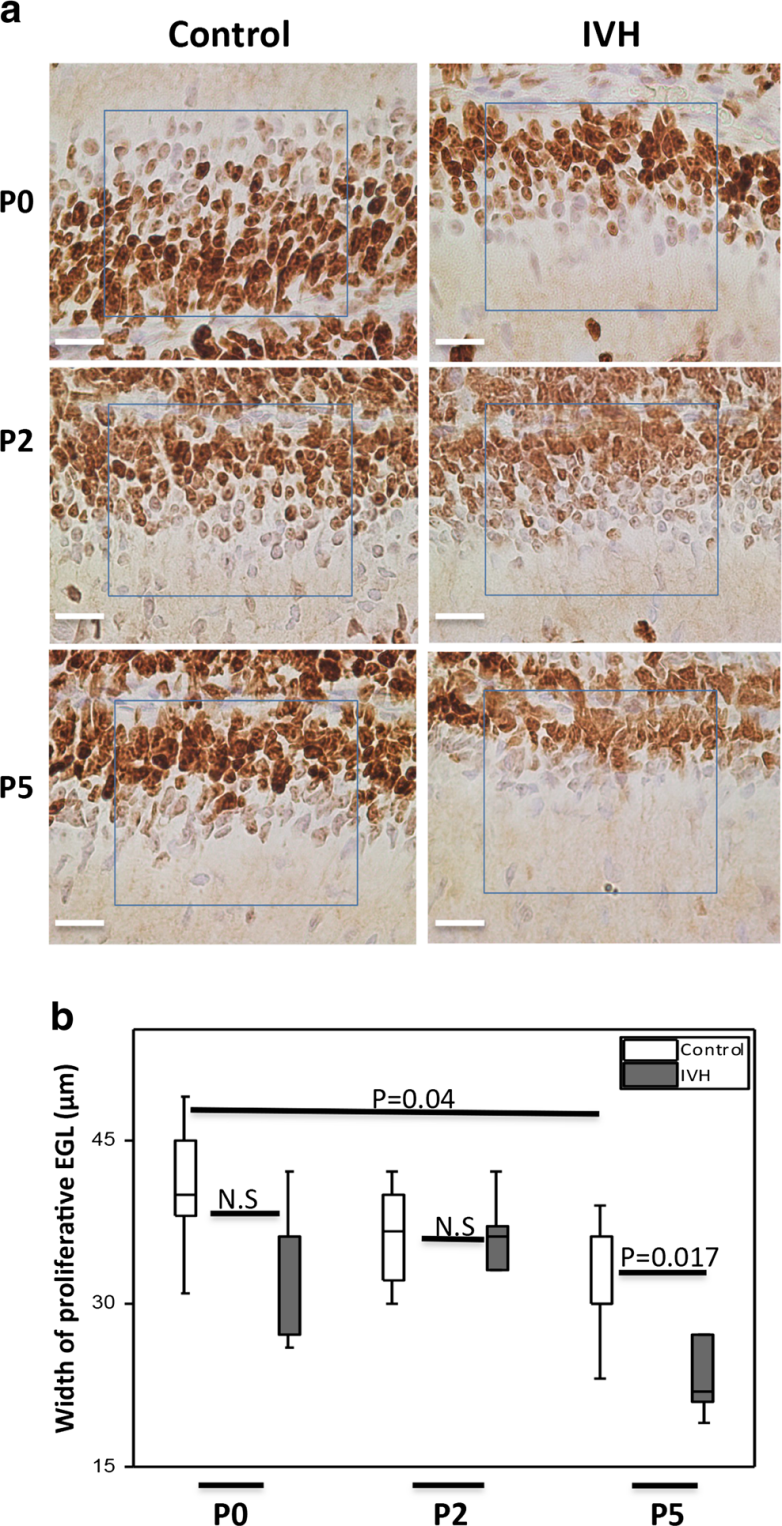
Reduced width of proliferative EGL following preterm IVH. **a** Images of EGL of the developing cerebellum from which quantitative measurements were made of the proliferative width in the respective groups at the time points studied. The image shows the Ki67-positive outer portion of the EGL where proliferation of granule cell precursors occurs and the deeper portion, which hosts the differentiation of granule cell precursors to mature granule cells. *Scale bar* = 50 μm. **b** GCP proliferation in the outer portion of the EGL of the developing cerebellum was investigated following IVH by Ki67 staining. Measurement of the width of proliferative EGL was done in cerebellar tissue sections of both sham controls (control, *white bars*; *n* at P0 = 6, *n* at P2 = 6, *n* at P5 = 5) and IVH pups (*dark gray bars*; *n* at P0 = 5, *n* at P2 = 6, *n* at P5 = 6) at P0, P2, and P5. Results are presented as box plots displaying medians and 25th and 75th percentiles. Statistical differences between groups for respective time points were analyzed using the Mann–Whitney *U* test

Measurement of the width (μm) of the proliferative external granular layer (EGL), as determined by Ki67-positive cells, was performed in four predefined regions. These regions were the inner and outer portions of lobule V and the inner and outer portions of lobule IX, respectively, as illustrated in Fig. 3 in the Data supplement. These regions were chosen because they represent regions with possible maturational differences in EGL proliferation and subsequent width. Measurements were performed with a bright-field microscope (Leica DMRX), using a ×40 dry objective lens. The average of the four respective measured widths was calculated for each pup.

Using the Leica Q500 image analysis system of the microscope, the areas of Iba1- and calbindin-positive stained cells were respectively determined in relation to the cerebellar white matter area and the area of the molecular layer. Thus, both positive Iba1 and calbindin staining were expressed as percentage positive area in relation to, respectively, a standardized area of the cerebellar white matter and of the molecular layer. Nonspecific background staining was taken into consideration with respect to a setup threshold.

For mRNA analysis, the rabbit pups were euthanized with intracardiac thiopental injection at P0. The brain was dissected out of the skull and cerebellar tissue collected, snap-frozen, and stored at −80 °C until further analysis as described below.

### RNA Isolation and Real-Time PCR

Total RNA was extracted from the cerebellar tissue of the rabbit pups using the NucleoSpin RNA/protein extraction kit as described by the manufacturer (Macherey-Nagel, Neumann-Neander, Düren, Germany). The optical density ratio (OD at 260 nm/280 nm) of extracted RNA samples was always approximately 2.0. Reverse transcription was performed according to the manufacturer’s instructions on 1 μg total RNA using iScript™ cDNA Synthesis Kit (Bio-Rad, Hercules, CA, USA). The RT^2^ qPCR Primer Assay (primer from QIAGEN, Germantown, MD, USA) was used to quantify mRNA expression of heme oxygenase 1 (HO-1), and expression was analyzed using iTaq Universal SYBR Green Supermix (Bio-Rad). Amplification was performed as described by the manufacturer (Bio-Rad) for 40 cycles in an iCycler Thermal Cycler (Bio-Rad), and data were analyzed using iCycler iQ Optical System Software (Bio-Rad). Data were normalized to glyceraldehyde-3-phosphate dehydrogenase (GAPDH, primer from QIAGEN), with fold change values calculated by normalizing against control animals.

### Statistics

Statistical analysis was performed with IBM SPSS Statistics version 22. Results are presented as medians (ranges) and displayed as box plots. Comparisons between unrelated groups were performed with the Mann–Whitney *U* test as appropriate. Comparisons between multiple groups were made using the Kruskal–Wallis test followed by pairwise comparison with significance values adjusted for multiple comparisons. *P* values <0.05 were considered significant.

## Results

### Extensive Presence of Cell-Free Hb in the Cerebellum Following IVH

Immunofluorescence labeling of Hb was evaluated at P0 and revealed extensive deposition of RBCs in the subarachnoid space surrounding the cerebellar lobuli following IVH, which was not observed in control animals (control and IVH in Fig. 2). Labeled Hb was widespread within the cerebellum and not associated with cell bodies (IVH in Fig. 2). Extensive deposition of radially oriented cell-free Hb was observed in the deeper cerebellar layers, in the molecular layer, and in the white matter. Relatively low amounts of cell-free Hb molecules were observed in the EGL and primarily in lobules in immediate proximity to large deposits of RBCs in the subarachnoid space.

To further investigate the indicated widespread presence of cell-free Hb in P0 IVH pups shown by immunofluorescence labeling, we performed RT-PCR analysis of mRNA expression in cerebellar tissue of the major heme-degrading protein heme-oxygenase 1 (HO-1). At P0, the HO-1 mRNA expression levels were tenfold higher in IVH pups compared to controls (Fig. 2m).

### EGL Proliferation Following IVH

The total width of the EGL comprises an outer proliferative portion where the granule cell precursors (GCPs) divide and a deeper portion where the granule cells differentiate [3]. The width of the outer proliferative (Ki67-positive) portion of the EGL was measured and compared between groups (Fig. 3a, b). The median (range) widths of the proliferative EGL were 36.0 (42–26), 36.0 (42–26), and 22.0 (27–19) μm, respectively, at P0, P2, and P5 in the IVH pups and 40.0 (49–31), 36.5(42–30), and 30.0 (39–23) μm, respectively, in the control pups. The median proliferative EGL width was significantly smaller in pups with IVH compared to control pups at P5 (*P* = 0.017) with a clear tendency at P0 (*P* = 0.08) (Fig. 3b).

### Purkinje Cell Maturation Following IVH

**Fig. 4:**
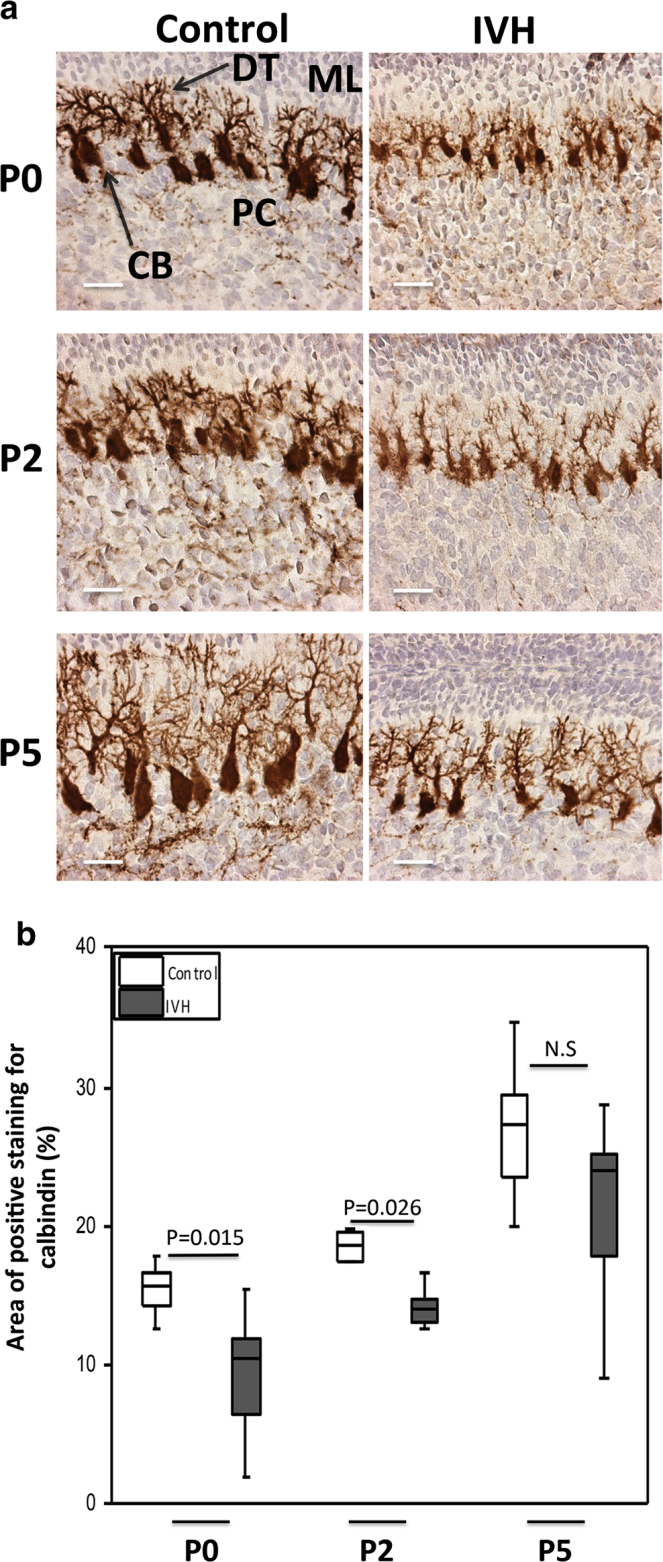
Impaired Purkinje cell maturation following preterm IVH. **a** Immunostaining of calbindin, a calcium-binding protein, was used as a marker of Purkinje cell development in the molecular layer of the developing cerebellum. Calbindin stains are seen as *brown* to *dark brown*. Decreased calbindin immunoreactivity was observed in IVH pups (*brown*) compared to controls (*intense dark brown*). Observation of neuronal morphology revealed smaller neuronal cell bodies and underdeveloped Purkinje dendrites in IVH pups compared to controls at postnatal time points of P0, P2, and P5. *ML* molecular layer, *PC* Purkinje cell, *DT* dendrites, *CB* cell bodies; *scale bar* = 50 μm. **b** Grading of Purkinje cell development by measurement of percentage area of positive calbindin staining was done in cerebellar tissue sections of both control (*white bars*; *n* at P0 = 6, *n* at P2 = 6, *n* at P5 = 5) and IVH pups (*dark gray bars*; *n* at P0 = 6, *n* at P2 = 6, *n* at P5 = 6) at P0, P2, and P5, as described in “Materials and Methods.” Results are presented as box plots displaying medians and 25th and 75th percentiles. Statistical differences between groups for respective time points were analyzed using the Mann–Whitney *U* test

Staining of calbindin, a calcium-binding protein, was used to evaluate Purkinje cell maturation in the molecular layer of the cerebellar cortex. IVH pups had smaller neuronal cell bodies and underdeveloped dendritic processes compared to control pups at P0, P2, and P5, respectively (Fig. 4a). Purkinje cell calbindin labeling was calculated and graded using densitometry, which showed that calbindin-labeled Purkinje cells at P0 and P2 had a significantly lower area in the IVH pups compared to controls (Fig. 4b; P0, *P* = 0.015; P2, *P* = 0.026); however, for smaller cells at P5, the differences were not statistically significant (*P* = 0.247). The smaller size in IVH animals indicated a reduced Purkinje cell differentiation and maturation in IVH animals.

### Microglial Response in Cerebellar White Matter Following IVH

**Fig. 5:**
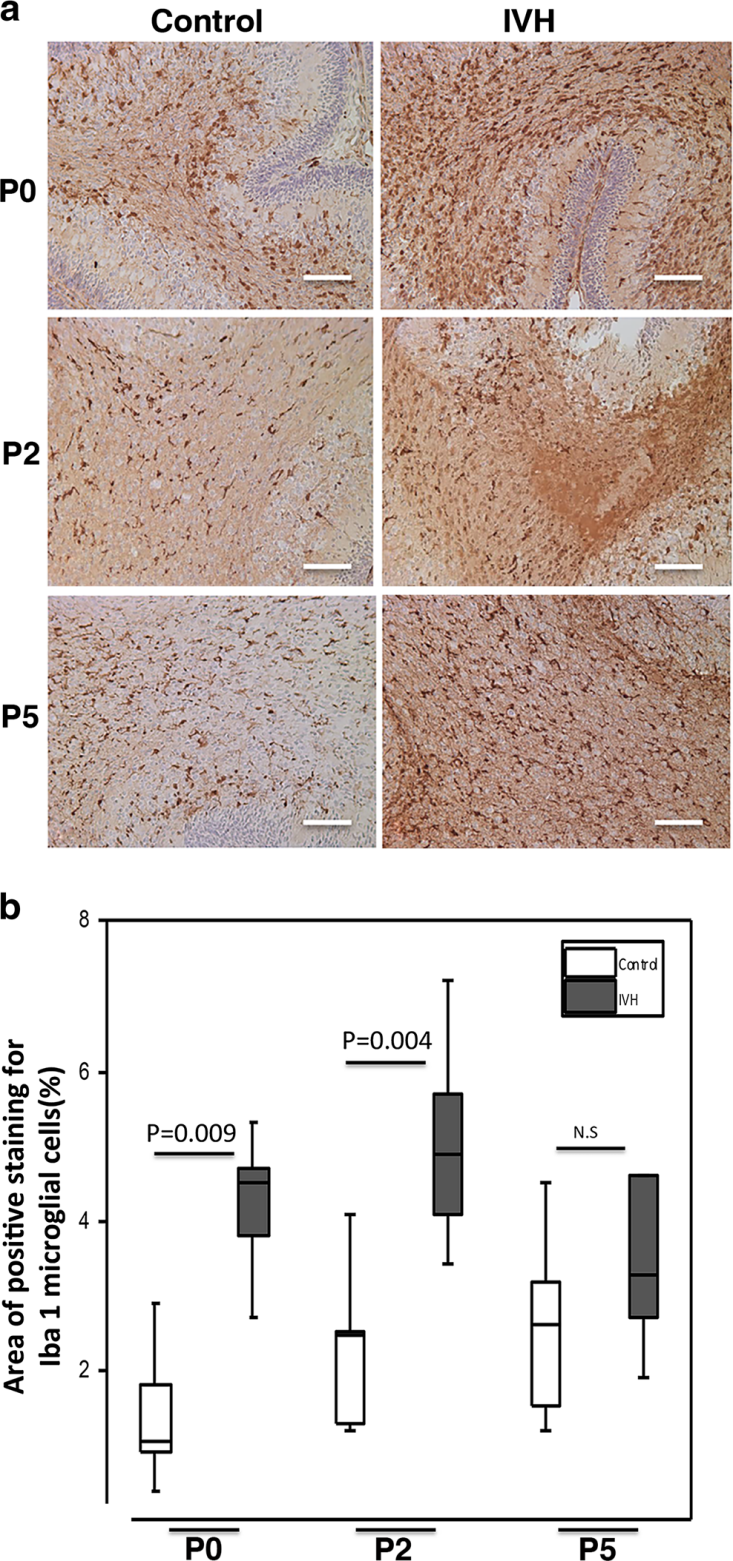
Microglial activation in the cerebellar white matter following preterm IVH. **a** Immunolabeling to confirm upregulation of Iba1 (seen as *brown* to *dark brown*) expression, a marker of microglial activation was used as a qualitative marker of reactive microglia cellular response in the white matter of the developing cerebellum. Increased Iba1 immunoreactivity was observed in IVH pups compared to controls at P0, P2, and P5. Observation of microglial morphology revealed an amoeboid shape with long processes in the IVH pups. *Scale bar* = 50 μm. **b** Measurement of percentage area of positive Iba1 staining was done in cerebellar tissue sections of both control (*white bars*; *n* at P0 = 6, *n* at P2 = 6, *n* at P5 = 5) and IVH pups (*dark gray bars*; *n* at P0 = 5, *n* at P2 = 6, *n* at P5 = 6) at P0, P2, and P5, as described in “Materials and Methods.” Results are presented as box plots displaying medians and 25th and 75th percentiles. Statistical differences between groups for respective time points were analyzed using the Mann–Whitney *U* test

Iba1 immunoreactivity was investigated to evaluate cerebellar white matter microglial response following IVH (Fig. 5a). At P0 and P2, IVH pups compared to control pups showed a significantly higher area of Iba1 immunoreactivity, based on cells with amoeboid morphology corresponding to activated microglia (P0, *P* = 0.009; P2, *P* = 0.004; see Fig. 5b). Microglial activation was less marked at P5 (*P* = 0.247) in both groups and did not differ significantly between groups.

### Hp Distribution Following Intraventricular Administration

At P0, the presence of Hp and its distributional relation to cell-free Hb was investigated in all groups by means of double immunofluorescence labeling (Fig. 2). Hp labeling was detected only in IVH pups that received intraventricular (human) Hp at 8 h of age. No Hp labeling was detected in control pups or in pups with IVH receiving only vehicle.

In IVH pups receiving human Hp, the Hp immunolabeling was widely distributed throughout large parts of the cerebellum. Double immunofluorescence labeling of Hp and Hb in these pups displayed a high degree of co-existence of human Hp and Hb in most regions, including the molecular layer and white matter (Fig. 2, IVH + Hp). Similar to labeling of cell-free Hb, labeling of Hp was relatively low in the EGL.

### Reduced Cerebellar Damage Following Hp Administration

**Fig. 6:**
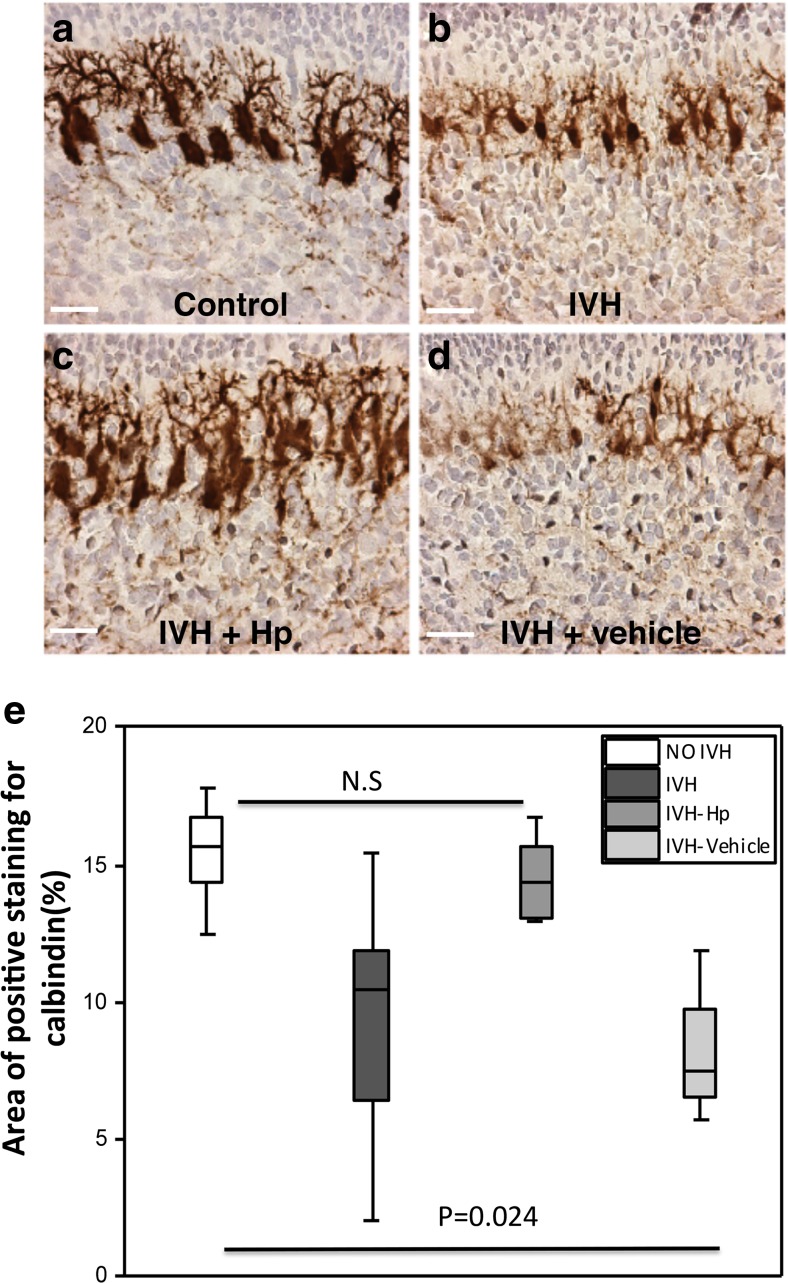
Intraventricular Hp administration protects against impaired Purkinje cell development following preterm IVH. **a**–**d** Following intraventricular Hp administration at P0, a higher intensity of calbindin immunoreactivity, relatively larger Purkinje cell bodies, and developed dendrites were observed in the Hp-administered IVH pups as compared to pups with IVH only or vehicle-treated IVH pups. *Scale bar* = 50 μm. **e** Grading of Purkinje cell development by measurement of percentage area of positive calbindin staining was done in cerebellar tissue sections at P0 of control pups (*white bars*, *n* = 6), IVH pups (*dark gray bars*, *n* = 6), and following intraventricular injection of Hp in pups with IVH (IVH + Hp, *gray bars*, *n* = 6) or vehicle solution (IVH + Vehicle, *light gray bars*, *n* = 4). Results are presented as box plots displaying medians and 25th and 75th percentiles. Differences between IVH + Hp vs. control and IVH + Vehicle vs. control were analyzed using the Kruskal–Wallis test followed by pairwise comparison with significance values adjusted for multiple comparisons

The group of pups receiving intraventricular administration of Hp following IVH (IVH + Hp), displayed an improved Purkinje cell maturation at P0 compared to both IVH + Vehicle pups and IVH pups (Fig. 6a–d). These findings included both a higher intensity of calbindin immunoreactivity and relatively larger neuronal cell bodies with more developed dendritic processes (Fig. 6a–d). Results from quantification of Purkinje cell development by calbindin staining densitometry showed an increased staining in the IVH pups following intraventricular Hp administration (Fig. 6e; Control, IVH + Hp, *P* = 1.00; Control, IVH + Vehicle, *P* = 0.024).

**Fig. 7:**
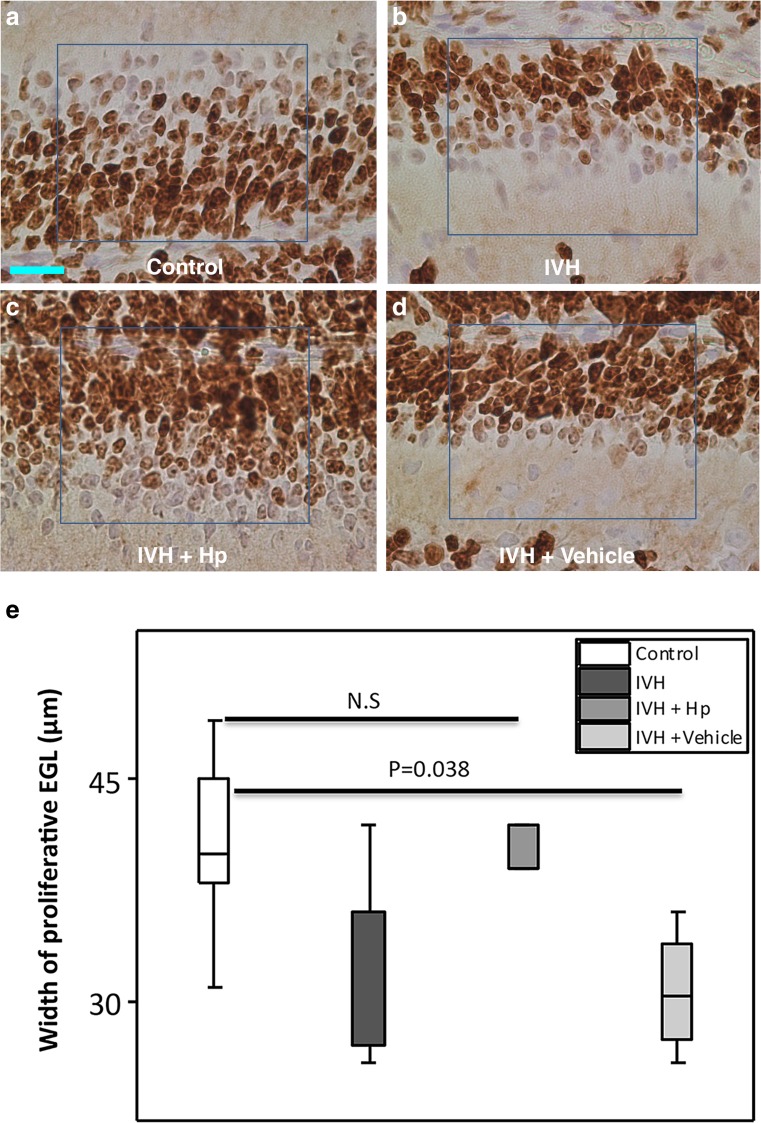
Intraventricular Hp administration protects against reduction in width of proliferative EGL following preterm IVH. **a**–**d** Following intraventricular Hp administration at P0, a higher intensity of Ki67 immunoreactivity was observed in the Hp-administered IVH pups as compared to pups with IVH only or vehicle-treated IVH pups. *Scale bar* = 20 μm. **e** Measurement of the width of Ki67-positive proliferative EGL was performed in cerebellar tissue sections at P0 of control pups (*white bars*, *n* = 6), IVH pups (*dark gray bars*, *n* = 5), and following intraventricular injection of Hp in pups with IVH (IVH + Hp, *gray bars*, *n* = 5) or vehicle solution (IVH + Vehicle, *light gray bars*, *n* = 4). Results are presented as box plots displaying medians and 25th and 75th percentiles. Differences between IVH + Hp vs. control and IVH + Vehicle vs. control were analyzed using the Kruskal–Wallis test followed by pairwise comparison with significance values adjusted for multiple comparisons

Furthermore, Hp administration restored the arrested cell proliferative activity in the outer portion of the EGL at P0 following IVH, as shown by the width of the proliferative part of the EGL in the respective treatment groups (described in Fig. 7a–d). The median (range) widths of the proliferative EGL were 39 (48–32) μm in the IVH + Hp pups, 30.5 (36–26) μm in the IVH + Vehicle pups, 36.0 (42–26) μm in the IVH pups, and 40.0 (49–31) μm in the control pups (Fig. 7e; Control, IVH + Hp, *P* = 0.93; Control, IVH + Vehicle, *P* = 0.038).

## Discussion

In this study, we show that IVH in the preterm rabbit pup is followed by an extensive deposition of blood products, specifically cell-free Hb, in the cerebellar cortex and white matter. This event is accompanied by a decrease in neuronal cell proliferation and a delay in Purkinje cell maturation. Intraventricular administration of the cell-free Hb scavenger Hp resulted in a high co-existence of administered Hp with cell-free Hb within the cerebellum. Furthermore, administered Hp partially reversed the cerebellar damage, indicating that cell-free Hb and its metabolites are causal in cerebellar underdevelopment. To the best of our knowledge, this work is the first animal study to evaluate cerebellar exposure to blood products and their role in cerebellar impairment following preterm IVH.

Following preterm IVH, there is a deposition of extravasated blood into the CSF of the intraventricular space. This deposition is followed by hemolysis of RBCs, leading to a release of cell-free Hb. Physiologically, cerebral CSF produced by the choroid plexus of the ventricular system passes through the fourth ventricle and enters the subarachnoid space, resulting in an immediate interface with the cortex of the developing cerebellum [27, 28]. Consequently, there is a strong physiological support for CSF containing extravasated blood reaching the cerebellum following cerebral IVH, as evidenced in this study by the visible presence of hemorrhagic CSF surrounding cerebellar tissue at termination of pups with IVH.

In the rabbit pup model, the spontaneous vessel rupture and the subsequent sequence of events leading to IVH mimics the situation in the human preterm infant quite well. It has been suggested that many of the effects observed in this model are related to the administered glycerol, including decreased proliferation leading to cerebellar hypoplasia [29]. Of importance in this study, as well as in our previous work, all pups including controls received the same dose of intraperitoneal glycerol, which rules out the possibility that the present findings in IVH pups are related to the administered glycerol.

Using Hb immunofluorescence and as demonstrated by autofluorescence, we identified an extensive deposition of RBCs and cell-free Hb in the subarachnoid space enveloping the cerebellar lobules following IVH. Cell-free Hb reached the innermost layers of the cerebellar cortex at P0 and was extensively deposited in the molecular layer and white matter of the cerebellum but to a much lesser extent in the EGL. Cell-free Hb within the EGL was basically found only in cerebellar lobules in immediate proximity to large deposits of RBCs in the subarachnoid space, possibly serving as a source of the cell-free Hb. In conjunction with the radial orientation of the Hb molecules, the high amount of cell-free Hb in the molecular layer and white matter suggests additional sources beyond the CSF in the subarachnoid space. Speculatively, the source of cell-free Hb could be via the roof of the fourth ventricle and transfer through the cerebellar peduncles to the white matter of the cerebellum.

Cell-free Hb and its metabolites, e.g., heme and iron, are well described to act as sources of ROS and free radicals, which are causal initiators of oxidative damage to cells and tissues [30]. We have previously shown that cell-free Hb and its metabolites, i.e., methemoglobin and heme, are potent inducers of pro-inflammatory pathways in choroid plexus epithelium and in astrocytes [17–19]. The extensive presence of cell-free Hb in the cerebellar white matter following IVH in this study was accompanied by clear signs of microglial activation in corresponding white matter regions, marked by increased expression of Iba1 antigen and an activated morphology in the IVH group (Fig. 5). This result suggests that deposited cell-free Hb may induce a microglial pro-inflammatory response with possible adverse effects on immature oligodendrocyte proliferation and maturation and subsequent cerebellar white matter damage. In the current study, using immunofluorescence and immunohistochemistry, we could not distinguish between different forms of oxidized Hb, e.g., oxyHb and metHb, and thus cannot conclude whether the effects observed are caused by oxyHb, metHb, or the downstream metabolites heme and iron.

Our finding that cell-free Hb is extensively deposited in the molecular layer of the cerebellum is a cause for concern because this layer constitutes the environment for Purkinje cell maturation. The Purkinje cells are intrinsically sensitive to oxidative stress and essential for establishing the cerebellar circuitry, which is vital for impulse transmission in the cerebellum [31–34]. In addition, mature Purkinje cells also play a vital role in the development of the EGL by sourcing GCPs with sonic hedgehog protein, an important mitotic growth factor vital to their proliferation [35, 36]. Consequently, exposure of the molecular layer to cell-free Hb not only will have neurotoxic effects on Purkinje cells but also will further impair the development of the EGL. The EGL of the developing cerebellum serves as a germinal center where GCPs proliferate and subsequently differentiate into mature granule cells. Granule cells are important for the structural integrity of the cerebellum; in addition, during their migration to form the granular layer, they transmit certain excitatory signals needed for the differentiation and maturation of the Purkinje cells. Thus, exposure of the developing cerebellum to cell-free Hb may lead to damaging effects not only to the cellular architecture but also to the functional integrity of the cerebellum, subsequently causing cerebellar underdevelopment.

To evaluate the possible effects of impaired Purkinje cell support and direct exposure to the hemorrhage, we performed metric analysis of Ki67 staining to evaluate EGL cell proliferation and thus pathological cellular senescence. Cellular senescence in this context can be seen as a process by which damage to tissue causes a decrease in metabolism leading to arrest of cell proliferation and recruitment of phagocytic immune cells to help in tissue renewal [37]. Measurements of the EGL (Fig. 3a) showed that IVH caused a significant decrease in the width of the proliferative portion of the EGL at P0 and P5 (Fig. 3b). This result is a clear indication that IVH-related processes cause impairment of the proliferative activity of the EGL. The postnatal time points P0 to P5 studied in the preterm rabbit pup correspond to the gestational ages of 25 to 35 weeks in humans, a period characterized by intense cell proliferation in the outer portion of the EGL [38]. In the human preterm infant, the width of the proliferative EGL decreases from 30 gestational weeks onwards as the GCPs mature into granule cells and leave the EGL to form the internal granular layer [3]. This timing corresponds well to our observations in the rabbit pup with EGL proliferative width in control pups showing a decrease in width from P0 to P5 (Fig. 3b).

Cell-free Hb may cause damage to the cerebellum in a number of different ways. Following hemolysis, release of excess cell-free Hb may lead to the formation of heme and free iron, increasing the concentration of redox-active iron in the extracellular environment. Both heme and free iron have a pro-oxidative damaging effect on cells, and iron overload has been reported to cause cerebral damage following IVH [39, 40]. Indeed, reduction in iron overload attenuated development of hydrocephalus and brain damage in a rodent model of neonatal germinal matrix hemorrhage [41]. In addition to its redox-related effects, cell-free Hb also acts as a redox-active damage-associated molecular pattern (known as DAMP) molecule that perturbs the innate immune homeostasis by triggering Toll-like receptor signal transduction pathways and causing pro-inflammatory damage to cells [42–44]. In this study, we investigated the causal importance of cell-free Hb in the impairment of Purkinje cell maturation and in the arrest of EGL cell proliferation by administering the Hb-scavenging protein Hp intraventricularly following detection of IVH. Hp binds to cell-free Hb, forming an inert Hb–Hp complex, which then channels the Hb molecules for intracellular degradation via CD163-mediated endocytosis [45, 46]. Intracellularly, the enzyme HO-1 breaks down heme to bilirubin and CO, both of which have antioxidant and vasodilatory benefits [47]. By forming a tight complex with cell-free Hb, Hp stabilizes and shields heme iron within the hydrophobic pocket of Hb, thereby preventing its cytotoxic and pro-oxidative effect [48]. The removal of cell-free Hb from the extracellular environment through its complex formation with Hp could thus reduce interaction with signal-transducing receptors of cells in the brain innate immune system and reduce exposure to excess iron and to heme-induced toxicity.

A neuroprotective role of induced endogenous Hp following intracerebral hemorrhage has been documented [49]. The induction of Hp was necessary because of very low levels of endogenous Hp in the human brain. In a previous study, the resting state capacity of the intrathecal Hb–Hp complex clearance was found to be 50,000-fold lower than that in the circulation in the adult. The system was quickly saturated during SAH with a residual inability to deal with cell-free Hb, clearly indicating an insufficient Hb scavenging capacity within the brain [50]. In view of this, we administered human Hp intraventricularly, which resulted in an extensive presence of Hp in the cerebellum. Hp was not detected in animals that did not receive exogenous Hp. The Hp labeling was specific for the administered human Hp, i.e., completely absent in sham-injected IVH pups as in IVH and control pups, thus excluding endogenous Hp as a source of the positive Hp labeling.

Our double immunofluorescence of Hb and Hp showed that the injected Hp reaches the same cerebellar areas as cell-free Hb and that the two are extensively co-localized in these regions. Hb and administered Hp co-existed in several regions of the cerebellum, mainly within the molecular layer and white matter and to a lesser degree in the EGL. Congruent with the anatomical co-existence of Hp and Hb, results showed that Hp administration partially reduced the Purkinje cell maturational arrest caused by IVH, represented by calbindin immunoreactivity showing a higher intensity of labeling, relatively larger cell bodies, and more extensive dendritic processes in pups receiving Hp as compared to the other IVH groups. Furthermore, Hp administration counteracted the decreased development of the proliferative region of the EGL following IVH and increased the proliferative width almost to the level of the control pups.

## Conclusion

In this study, we showed that IVH in the preterm rabbit pup is followed by an extensive deposition of cell-free Hb in cerebellar cell layers and white matter. This exposure to cell-free Hb was associated with microglial activation, an arrest in neuronal cell proliferation, and a delayed Purkinje cell maturation. Intraventricular administration of the cell-free Hb scavenger Hp partially blocked these effects, suggesting that cell-free Hb and its downstream metabolites are causal in cerebellar impairment following IVH. In terms of future clinical application, these results suggest that removal or scavenging of Hb metabolites following IVH, for instance by administered Hp, may reduce subsequent cerebellar impairment.

BSA, bovine serum albumin; EGL, external granular layer; GAPDH, glyceraldehyde-3-phosphate dehydrogenase; GCP, granular cell precursor; Hb, hemoglobin; Hp, haptoglobin; IVH, intraventricular hemorrhage; PBS, phosphate buffer saline; PFA, paraformaldehyde; SAH, subarachnoid hemorrhage

## Electronic supplementary material

Supplementary Fig. 1 Antibody specificity of the immunofluorescence labeling of Hb and Hp. Antibody specificity tests on cerebellar sections from rabbit pups showed that the immunofluorescence labeling is the result of specific binding of the endogenous rabbit Hb and administered human Hp (i.e., not endogenous) to their corresponding epitopes (see Fig. 2). This inference is further supported by the lack of Hb labeling in control animals and of Hp labeling in control animals as well as in IVH animals that did not receive injections of human Hp (i.e., no labeling of endogenous Hp). These tests also showed that the endogenous tissue fluorescence could be concluded to arise only from cell bodies, preferentially from whole erythrocytes located in the arachnoid space (arrows in B and D), which was even more pronounced in IVH animals (see Fig. 2). Thus, the detected extracellular Hb and Hp can be considered to represent a specific detection and visualization of their distribution in the cerebellum. The antibody control sections were processed for double immunofluorescence labeling (see also Fig. 2) with the only difference that the primary antibody incubation was excluded from the protocol (i.e., no anti-Hb or anti-human Hp antibodies). Antibody specificity control sections were used in every labeling experiment to eliminate the risk for false interpretation of fluorescence caused by nonspecific secondary antibody binding or endogenous cell/tissue autofluorescence. These sections were also used during the analyses to ensure that the “threshold” for the fluorescence detection level visualized only immunofluorescence from secondary antibodies bound to anti-Hb and anti-human Hp antibodies, i.e., not background levels of fluorescence from nonspecific binding of secondary antibodies. The images show a representative section from an animal with IVH that received Hp injection. Nuclear counterstaining was performed with DAPI (blue in A and D). D is a merged image from all visualized channels (A–C) of DAPI together with the fluorophore visualization used for Hb (red) and Hp (green) immunofluorescence of the used secondary antibodies targeting the anti-rabbit Hb and injected anti-human Hp (B and C). B and C show the lack of binding by the used secondary antibodies to cell bodies or other extracellular targets comparable to their presented targeting of our anti-rabbit Hb and anti-human Hp antibodies. Thus, the immunofluorescence labeling presented is most likely due to secondary antibody binding to the primary antibodies, which bind to the rabbit Hb and human Hp epitopes, respectively. 20 μm (GIF 158 kb)

High resolution image (TIFF 24909 kb)

Supplementary Fig. 2 Antibody specificity of the immunohistochemical labeling of calbindin, Ki67, and Iba1. No immunolabeling or background staining was observed in sections when primary antibodies were omitted from the immunohistochemical labeling protocol (A–D). Images illustrate the staining with only anti-mouse secondary antibodies conjugated with BrightVision-HRP, used for calbindin and Ki67 labelings, in a P5 control animal (A) and in a rabbit pup with IVH (B). C and D illustrate staining achieved when using the anti-rabbit secondary antibodies conjugated with BrightVision-HRP for Iba1 labelings, in a P5 control animal (C) and a P5 rabbit pup with IVH (D). Scale bar = 50 μm (GIF 447 kb)

High resolution image (TIFF 24909 kb)

Supplementary Fig. 3 An overview of the cerebellar lobuli. The image is a pictorial representation of the cerebellar lobules used for the EGL analysis. It shows the four predefined regions from which the metric analysis of the width of the proliferative EGL was done. These regions were the inner (designated as in) and outer portions (designated as out) of lobule V EGL germinal region and the inner (designated as in) and outer portions (designated as out) of lobule IX EGL germinal region. Scale bar = 50 μm (GIF 367 kb)

High resolution image (TIFF 24909 kb)
