# Supplementary figures and images for: Cerebellar Exposure to Cell-Free Hemoglobin Following Preterm Intraventricular Hemorrhage: Causal in Cerebellar Damage?
Source: Transl Stroke Res. 2017 Jun 10;8(5):461–73. doi: 10.1007/s12975-017-0539-1 (PMC5590031; doi:10.1007/s12975-017-0539-1)

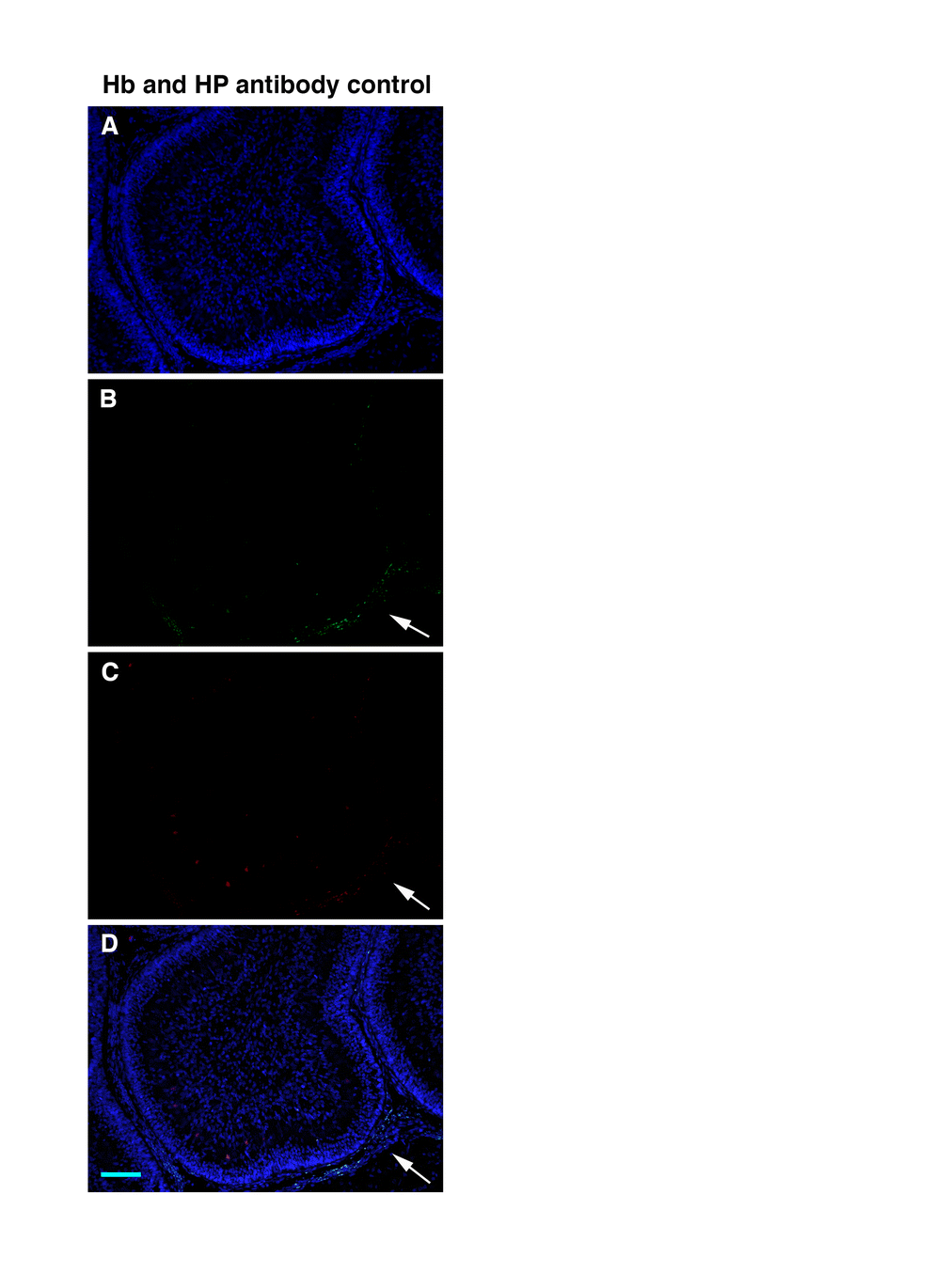

Supplement: Supplementary file 1 — Antibody specificity of the immunofluorescence labeling of Hb and Hp. Antibody specificity tests on cerebellar sections from rabbit pups showed that the immunofluorescence labeling is the result of specific binding of the endogenous rabbit Hb and administered human Hp (i.e., not endogenous) to their corresponding epitopes (see Fig. 2). This inference is further supported by the lack of Hb labeling in control animals and of Hp labeling in control animals as well as in IVH animals that did not receive injections of human Hp (i.e., no labeling of endogenous Hp). These tests also showed that the endogenous tissue fluorescence could be concluded to arise only from cell bodies, preferentially from whole erythrocytes located in the arachnoid space (arrows in B and D), which was even more pronounced in IVH animals (see Fig. 2). Thus, the detected extracellular Hb and Hp can be considered to represent a specific detection and visualization of their distribution in the cerebellum. The antibody control sections were processed for double immunofluorescence labeling (see also Fig. 2) with the only difference that the primary antibody incubation was excluded from the protocol (i.e., no anti-Hb or anti-human Hp antibodies). Antibody specificity control sections were used in every labeling experiment to eliminate the risk for false interpretation of fluorescence caused by nonspecific secondary antibody binding or endogenous cell/tissue autofluorescence. These sections were also used during the analyses to ensure that the “threshold” for the fluorescence detection level visualized only immunofluorescence from secondary antibodies bound to anti-Hb and anti-human Hp antibodies, i.e., not background levels of fluorescence from nonspecific binding of secondary antibodies. The images show a representative section from an animal with IVH that received Hp injection. Nuclear counterstaining was performed with DAPI (blue in A and D). D is a merged image from all visualized channels (A–C) of [file 12975_2017_539_Fig8_ESM.gif]

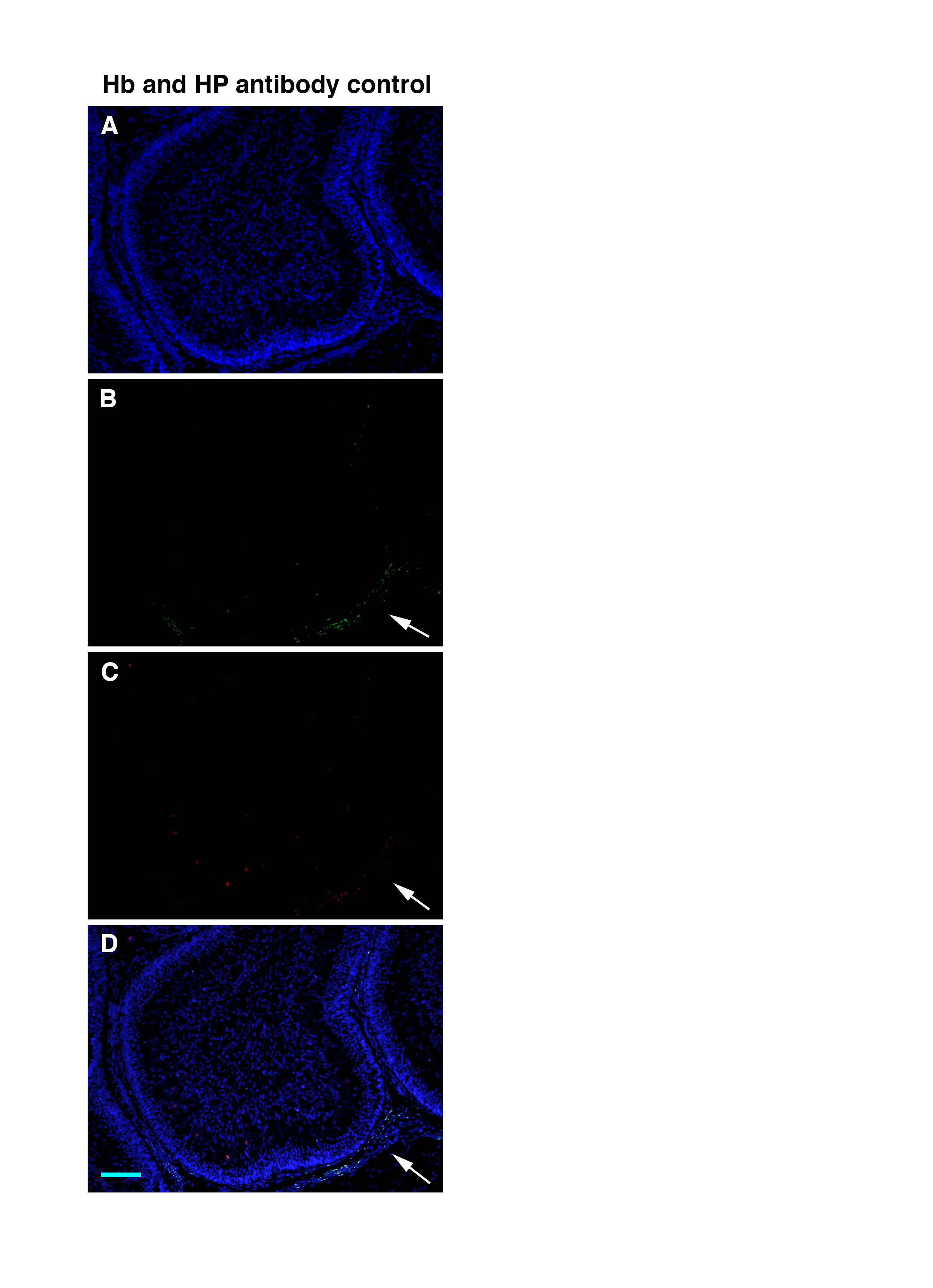

Supplement: Supplementary file 2 — High resolution image (TIFF 24909 kb) [file 12975_2017_539_MOESM1_ESM.tif]

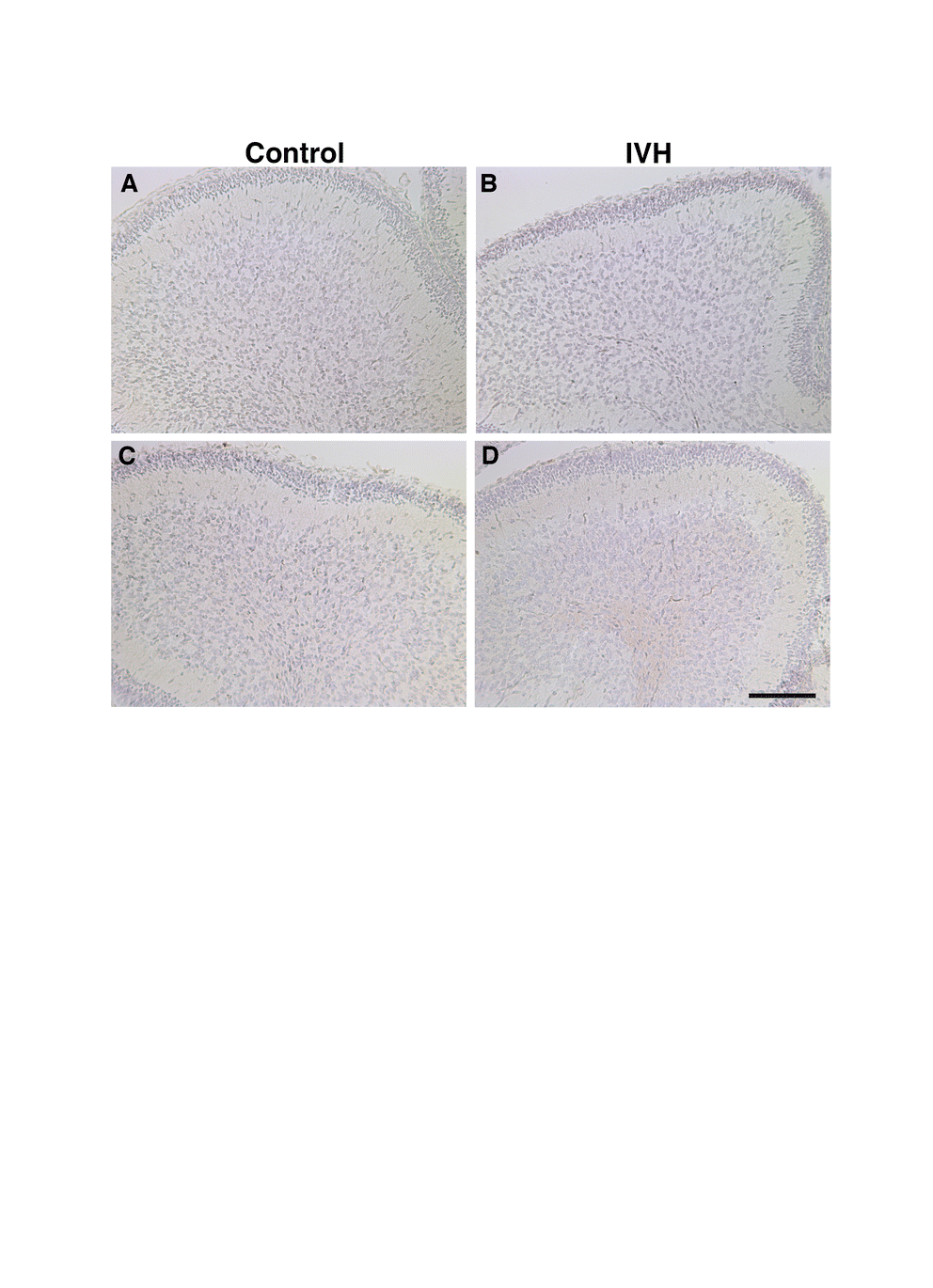

Supplement: Supplementary file 3 — Antibody specificity of the immunohistochemical labeling of calbindin, Ki67, and Iba1. No immunolabeling or background staining was observed in sections when primary antibodies were omitted from the immunohistochemical labeling protocol (A–D). Images illustrate the staining with only anti-mouse secondary antibodies conjugated with BrightVision-HRP, used for calbindin and Ki67 labelings, in a P5 control animal (A) and in a rabbit pup with IVH (B). C and D illustrate staining achieved when using the anti-rabbit secondary antibodies conjugated with BrightVision-HRP for Iba1 labelings, in a P5 control animal (C) and a P5 rabbit pup with IVH (D). Scale bar = 50 μm (GIF 447 kb) [file 12975_2017_539_Fig9_ESM.gif]

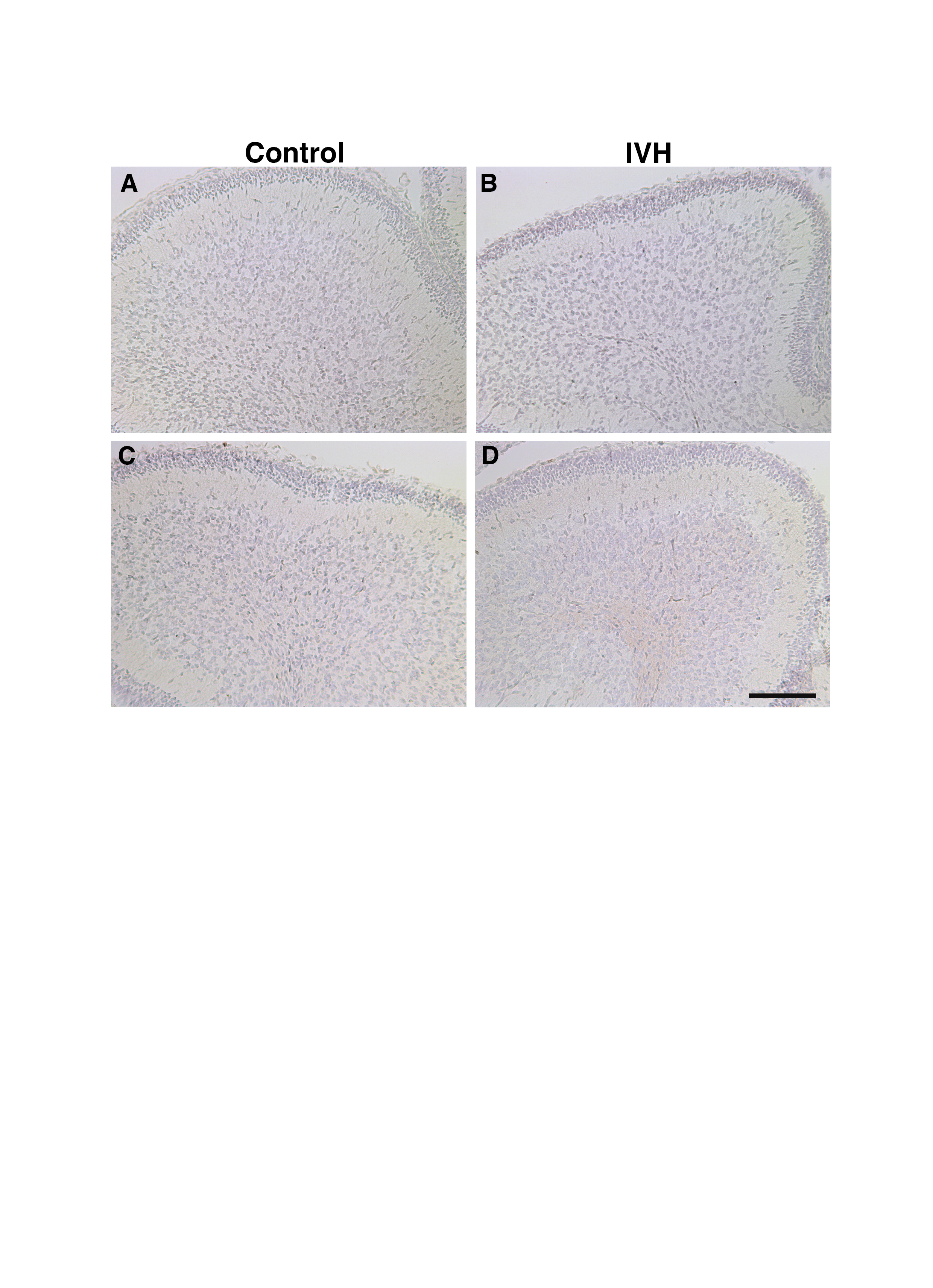

Supplement: Supplementary file 4 — High resolution image (TIFF 24909 kb) [file 12975_2017_539_MOESM2_ESM.tif]

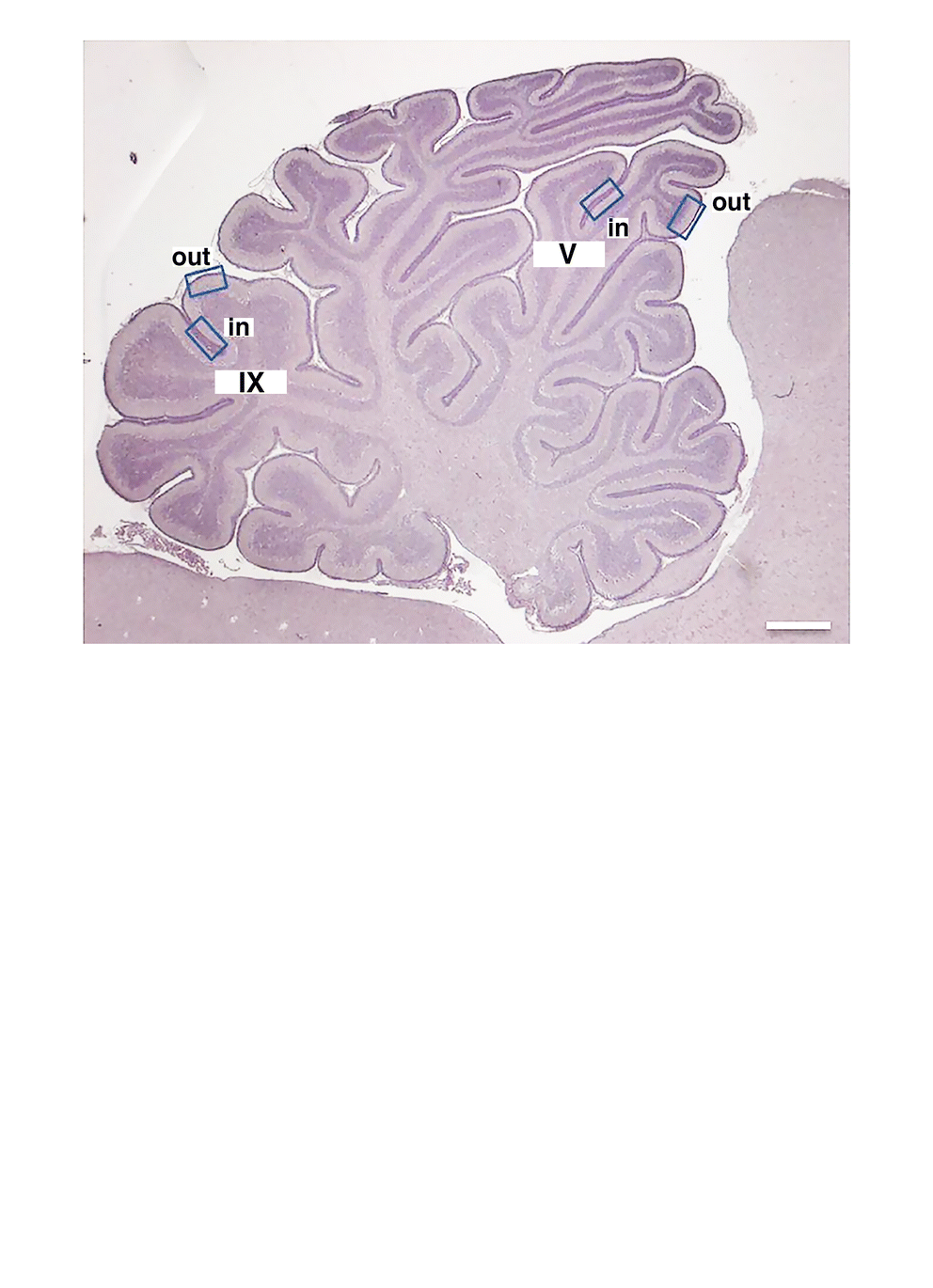

Supplement: Supplementary file 5 — An overview of the cerebellar lobuli. The image is a pictorial representation of the cerebellar lobules used for the EGL analysis. It shows the four predefined regions from which the metric analysis of the width of the proliferative EGL was done. These regions were the inner (designated as in) and outer portions (designated as out) of lobule V EGL germinal region and the inner (designated as in) and outer portions (designated as out) of lobule IX EGL germinal region. Scale bar = 50 μm (GIF 367 kb) [file 12975_2017_539_Fig10_ESM.gif]

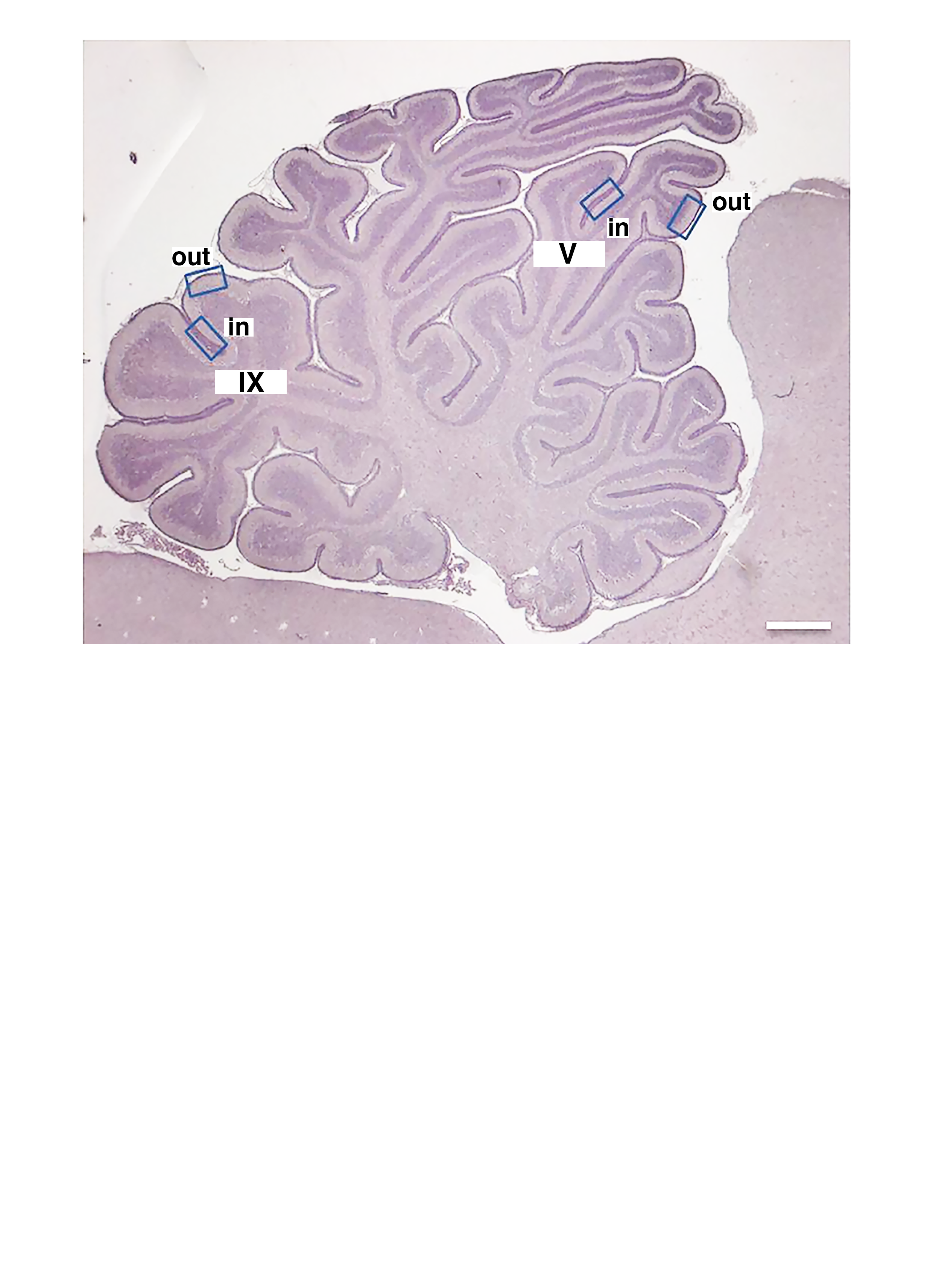

Supplement: Supplementary file 6 — High resolution image (TIFF 24909 kb) [file 12975_2017_539_MOESM3_ESM.tif]
